# First report of adult *Hyalomma marginatum rufipes* (vector of Crimean-Congo haemorrhagic fever virus) on cattle under a continental climate in Hungary

**DOI:** 10.1186/1756-3305-5-170

**Published:** 2012-08-13

**Authors:** Sándor Hornok, Gábor Horváth

**Affiliations:** 1 Department of Parasitology and Zoology, Faculty of Veterinary Science, Szent István University, Budapest, Hungary; 2 Veterinary Authority, Nagyatád, Hungary

**Keywords:** *Hyalomma marginatum rufipes*, Cattle, Continental climate, Crimean-Congo haemorrhagic fever

## Abstract

**Background:**

South Hungary is being monitored for the northward spreading of thermophilic ixodid species, therefore ticks were collected from cattle and wild ruminants (red, fallow and roe deer) in the autumn of 2011.

**Findings:**

Besides indigenous species (1185 *Dermacentor reticulatus* and 976 *Ixodes ricinus*), two *Hyalomma marginatum rufipes* males were found on two cows, in September eight days apart.

**Conclusions:**

This is the northernmost autochthonous infestation of the type host (cattle) with *H. m. rufipes*, vector of Crimean-Congo haemorrhagic fever virus. The present findings are suggestive of the moulting success of this Afro-Mediterranean tick species in a continental climate in Central Europe.

## Background

Within Central Europe Hungary is especially suitable for studying the northward spreading of thermophilic tick species, since it lacks high mountain ranges present along the same latitude in surrounding countries (i.e. the Alps and the Carpathians). Concerning environmental factors that influence tick distribution, south-western Hungary has the highest mean winter temperature (above −1°C) and precipitation (above 800 mm per year) in the country. Here, along the Croatian side of the border, Mediterranean ixodid species are known to occur [1]. In addition, a locally warmer continental climate may also allow future establishment of ticks transported by migratory birds from even larger distances [2]. Therefore it was decided to monitor south Hungary for emerging tick species. The study area was selected by taking into account places of cattle keeping, game reserves and the most likely future occurrence of *Hyalomma marginatum* in Hungary according to prediction models [3].

**Figure 1:**
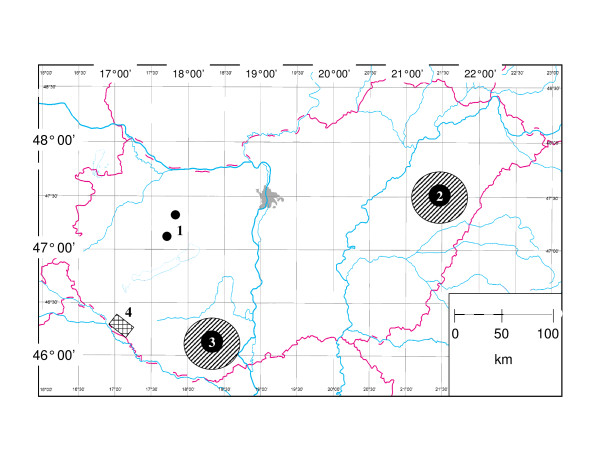
**Map of Hungary showing in chronological order regions for: (1) CCHF virus isolation in 1972, (2) autochthonous bovine and ovine CCHF seropositivities in 1973, (3) one human CCHF case in 2004; and (4) the study area.** Large, ruled circles indicate potential areas for infectious source.

Ticks were collected during the autumn (September-November) of 2011 from 20 beef cattle grazing outside, as well as from fresh carcasses of 82 red deer (*Cervus elaphus*), 20 fallow deer (*Dama dama*) and 6 roe deer (*Capreolus capreolus*) in south-western Hungary. All evaluated cattle were raised locally, and never left the country. Their contact with game animals can be excluded. The study area measured 20 × 30 km and is found at geographical coordinates 46° 20' N, 17° 10' E (Figure 1: site 4). All specimens of ticks were removed by forceps and put into 70% ethanol. Species were identified according to standard keys [4].

## Findings

**Figure 2:**
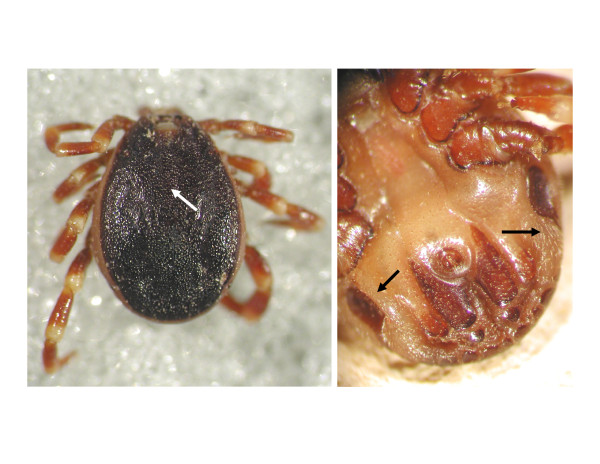
**Key features for the identification of *H. m. rufipes* males in the present study.** Arrows indicate dense punctuations dorsally (left) and setae around the spiracles ventrally (right).

A total of 2163 adult ticks were collected from cattle and wild ruminants. The majority of ticks from cattle were *Dermacentor reticulatus* (442 of 524: 84%), with a lower abundance of *Ixodes ricinus* (80 of 524: 15%). The proportion of these two species was more equilibrated on game animals (743 of 1639: 45% *D. reticulatus*, 896 of 1639: 55% *I. ricinus*). Apart from indigenous tick species, two *Hyalomma marginatum rufipes* males were also found on two cows, at the end of September eight days apart. These ticks were identified by the apparent dense dorsal punctuation and setae around the spiracles (Figure 2). To the best of our knowledge, this is the first recent report of infestation with adults of any *Hyalomma* spp. – in the same area, on two occasions – outside their known geographical range in Europe.

The main host of adult *H. m. rufipes* is cattle, but wild ungulates are also frequently infested [4]. However, during the present survey this tick species was absent from game animals. One factor that may have contributed to this is that *Hyalomma* spp. prefer open country habitats [5], therefore are more likely to attach to cattle which permanently graze on pastures. Similarly, *D. reticulatus* as an open country tick species [5] was significantly (Fisher’s exact test: P < 0.0001) more prevalent on cattle during the present survey, than on game animals partly with forested habitats.

*H. m. rufipes* is the most widespread species of the genus in Africa, but decades-old data attest to its occurrence along the northern latitude of Hungary in east Europe (including Russia: Caspian Sea region, Ukraine: Black Sea region), and to the south in Balkan countries such as Romania, Serbia, Bosnia-Herzegovina and Macedonia [6-8]. The most likely scenario for *H. m. rufipes* found on occasions in any part of Europe is importation of immature stages by migratory birds, arriving during the spring from the south [7].

Consequently, adults of this tick species are expected to be most active during the summer, as reported in Romania south-east of Hungary [6]. However, during an extensive tick collection in the study area during the preceding summer no *Hyalomma* specimens were found [9]. A plausible explanation for finding *H. m. rufipes* adults in the autumn is that cumulative temperatures are lower in Hungary, than in regions towards the south where *H. m. rufipes* is indigenous. In such conditions *Hyalomma* nymphs engorging at the time of migratory bird arrival in the spring may need much longer to moult [10].

In Europe *H. m. rufipes* subadults collected from migratory birds were reported several times, up to the northern latitude of Norway [11]. However, accounts of the occurrence of adult *Hyalomma* individuals north of the Mediterranean basin are scarce. One *H. m. rufipes* male was reported from a horse in an oceanic climate in the Netherlands [12], and one female *H. lusitanicum* also from a horse in a continental climate in Germany [13]. The origin of another, questing *H. m. marginatum* in Germany was reported to be doubtful [13]. The establishment of *Hyalomma* populations in the north are limited by factors (most notably by cumulative autumn temperatures) that affect moulting to the adult stage, and not by extremely cold winter temperatures which can be tolerated by post-moult adults [10]. In particular, the geographical range of *H. m. rufipes* can extend to areas with up to 120 days of frost annually [14]. Consequently, even small local populations may establish from nymphs dispersed by migrating birds, as reported in Russia [14].

The most severe risk posed by the presence of *Hyalomma* spp. is the transmission of the zoonotic Crimean-Congo haemorrhagic fever (CCHF) virus for which they are considered to be the primary vectors [15]. To be specific, epidemiological studies showed that *H. m. rufipes* may harbour different genotypes of CCHF virus naturally, and in certain regions this tick subspecies is thought to play a leading role in the maintenance of CCHF endemicity [16,17]. As demonstrated experimentally, *H. m. rufipes* is able to take up, to maintain (both transstadially and transovarially) and to inoculate CCHF virus [18-20].

It was postulated that − based on the rare emergence of *Hyalomma* adults − future outbreaks of CCHF will involve Western Europe [21]. Accordingly, the sporadic appearance of exotic *Hyalomma* spp. in Hungary may also be relevant to the fact that autochthonous and northernmost occurrence of CCHF in Central Europe was reported from Hungary [22]. In particular, the virus was isolated in 1972 [23] and one person contracted the disease in 2004 [24] in counties of Hungary next to the one where *H. m. rufipes* was presently found (Figure 1: sites 1,3). In 1973 seropositivity to CCHF virus was also detected in aboriginally Hungarian cattle and sheep [25] (Figure 1: site 2).

## Conclusions

Infestations of cattle with adults of *H. m. rufipes*, vector of CCHF virus, were recorded for the first time in Central Europe. The present findings are suggestive of the moulting success of *Hyalomma* nymphs (transported by migratory birds) under continental climate. Since *Hyalomma* adults were collected in an area predicted to have a higher risk for the future establishment of *H. marginatum*[3], data reported here also highlight the importance of predictive models. At the same time, these results support the need for continuous tick surveillance in similar regions, where exotic/emerging tick species are more likely to occur, which should be part of an integrated approach to monitor vector-borne diseases in Europe [26].

## Abbreviation

CCHF, Crimean-Congo haemorrhagic fever.

## Competing interests

The author(s) declare that they have no competing interests.

## Authors’ contributions

SH collected ticks and identified tick species, wrote the manuscript. GH also collected ticks. Both authors read and approved the manuscript.
